# From reactive to resilient: leveraging artificial intelligence for pandemic preparedness amid converging global threats

**DOI:** 10.1016/j.lana.2026.101533

**Published:** 2026-06-15

**Authors:** Attila J. Hertelendy, Nikki Romanik, Gregory R. Ciottone

**Affiliations:** a Department of Information Systems and Business Analytics, College of Business, Florida International University, Miami, FL, USA; b Disaster Medicine Fellowship, Department of Emergency Medicine, Beth Israel Deaconess Medical Center, Boston, MA, USA; c Georgetown / Medstar Health National Center for Health Security and Resilience, Washington, DC, USA; d Georgetown University Medical Center, Washington, DC, USA; e Harvard Medical School, Boston, MA, USA

Two infectious agent outbreaks unfolding in May 2026 expose the fragility of pandemic preparedness. On 2 May, the World Health Organization (WHO) was notified of a hantavirus cluster aboard the MV Hondius, an Antarctic expedition cruise; by 8 May, Andes virus had been reported in eight cases, six of whom were laboratory confirmed, and three of whom died and contact tracing extended across 23 countries.^1^ By 26 May, the total had reached 13 cases (11 laboratory confirmed, 2 probable; case fatality ratio 23%).^2^ Fifteen days later the WHO Director-General declared a Public Health Emergency of International Concern for an Ebola outbreak caused by Bundibugyo virus in north-eastern Democratic Republic of the Congo (DRC) and Uganda. This is DRC's 17th Ebola outbreak since 1976, in a region without licensed vaccines or therapeutics for this species and amid security, displacement, and cross-border trade concerns.^3^ These events come five years after The Independent Panel for Pandemic Preparedness and Response warned that, absent structural reform, COVID-19 would not be the last pandemic to find the world unprepared.^4^

The recurring failures remain the same. Detection continues to be slow: the MV Hondius index patient was symptomatic for weeks before laboratory confirmation, and in Bunia, initial negative results for Zaire Ebola virus delayed recognition of the Bundibugyo strain by weeks^5^ allowing undetected spread into Goma and Kampala. Compounding this inadequacy, response remains fragmented: genomic, clinical, and event-based signals persist in separate systems, on separate timelines, with separate gatekeepers. Zoonotic spillover events are increasing in frequency and originate disproportionately in wildlife,^6^ yet global surveillance investment remains poorly aligned with those geographies, and the potential for surge capacity in the Americas, where 229 hantavirus cases and 59 deaths were reported across eight countries in 2025, has not kept pace.^7^

Technology is the differentiator capable of compressing these timelines. Integration of predictive analytics, artificial intelligence (AI), and geospatial intelligence into clinical command systems has been proposed as a foundational capability for disaster response since the early COVID-19 wave^8^; six years later, that integration remains aspirational rather than operational. Three capabilities warrant urgent investment.

First, AI-enabled event-based intelligence, must be embedded within regional ministries of health rather than confined to academic and commercial silos, and powered by large-language-model pipelines that ingest multilingual clinical, veterinary, and media data in near real time.

Second, wastewater and environmental metagenomics provide a sentinel layer for both known and unknown pathogens of pandemic potential, with sensitivity that precedes clinical recognition by days to weeks; the architecture is feasible but remains under-deployed across both high-income and low- and middle-income countries.^9^ Had wastewater metagenomic surveillance been operational, it may have provided an earlier indication of Andes virus circulation before the MV Hondius cluster crossed the threshold for clinical recognition.^10^

Third, the epidemiological value of environmental metagenomics is amplified when integrated with syndromic surveillance. A modern biological intelligence infrastructure would combine metagenomically sequenced wastewater, syndromic surveillance, and behavioral indicators such as over the counter and prescription medication usage trends. The critical capability gap is not the absence of dashboards, but the absence of integrated analytics capable of detecting anomalous patterns suggestive of emerging outbreaks.^9^^,^^11^

These capabilities exist; the obstacle is not technology, but architecture. The structural barrier to achieving this integration is not technological capacity but design philosophy. During COVID-19, the absence of a unified analytical layer forced decision-makers to synthesize signals from multiple disconnected surveillance streams.

Many of the COVID-era surveillance advances are now being eroded: at the time of writing, the CDC's National Wastewater Surveillance System (NWSS) and Traveler Based Genomic Sequencing programs were transitioning from emergency supplemental funding which ends at the end of the current fiscal year to uncertain long-term sustainable financing.

These technologies do not replace clinical surveillance, field epidemiology, or trust-based community engagement; none will be effective without those foundations. But the next pandemic will not wait for human-paced systems to catch-up. The Americas region has digital infrastructure, and the scientific capacity traffic patterns that make it both highly exposed and uniquely positioned to lead. The choice is whether technology is treated as a peripheral enhancement or as the central architecture of the next response. Hantavirus aboard the MV Hondius and Bundibugyo Ebola in Ituri province are reminders that this question is no longer hypothetical.

## Contributors

AJH conceptualized the manuscript and produced the first draft. NR and GRC critically revised the manuscript for important intellectual content. All authors approved the final version.

## Declaration of interests

NR has declared the following financial interests: Council on Strategic Risks, Payment for speaking roles. Nevada Public Health Association, payment for travel to attend meeting and provide keynote address, Bowling Green State University, Payment for travel and keynote address. NR declared a non financial interest as advisor, 20/20 Network.
